# The Italian tremor Network (TITAN): rationale, design and preliminary findings

**DOI:** 10.1007/s10072-022-06104-w

**Published:** 2022-05-24

**Authors:** Roberto Erro, Andrea Pilotto, Marcello Esposito, Enrica Olivola, Alessandra Nicoletti, Giulia Lazzeri, Luca Magistrelli, Carlo Dallocchio, Roberta Marchese, Matteo Bologna, Alessandro Tessitore, Salvatore Misceo, Angelo Fabio Gigante, Carmen Terranova, Vincenzo Moschella, Lazzaro di Biase, Raffaella Di Giacopo, Francesca Morgante, Francesca Valentino, Anna De Rosa, Assunta Trinchillo, Maria Chiara Malaguti, Livia Brusa, Angela Matinella, Francesca Di Biasio, Giulia Paparella, Rosa De Micco, Elena Contaldi, Nicola Modugno, Alessio Di Fonzo, Alessandro Padovani, Paolo Barone, TITAN Study Group

**Affiliations:** 1 Department of Medicine, Surgery and Dentistry “Scuola Medica Salernitana”, Neuroscience Section, University of Salerno, Via Allende 43, 84081 Baronissi, SA Italy; 2 Neurology Unit, Department of Clinical and Experimental Sciences, University of Brescia, Brescia, Italy; 3 Clinical Neurophysiology Unit, AORN Cardarelli, Naples, Italy; 4 Neuromed Institute IRCCS, Pozzilli, IS Italy; 5 Department “G.F. Ingrassia”, Section of Neurosciences, University of Catania, Catania, Italy; 6 Neurology Unit, Department of Neuroscience, Dino Ferrari Center, Fondazione IRCCS Ca’ Granda Ospedale Maggiore Policlinico, Milan, Italy; 7 Department of Translational Medicine, Movement Disorders Centre, Neurology Unit, University of Piemonte Orientale, Novara, Italy; 8 Neurology Unit, Department of Medical Area, ASST Pavia, Voghera, PV Italy; 9 IRCCS Ospedale Policlinico San Martino, Genoa, Italy; 10 Department of Human Neurosciences, Sapienza University of Rome, Rome, Italy; 11 Department of Advanced Medical and Surgical Sciences, Università Della Campania “Luigi Vanvitelli”, Naples, Italy; 12 Neurosensory Department, Neurology Unit, San Paolo Hospital, ASL Bari, Bari, Italy; 13 Department of Clinical and Experimental Medicine, University of Messina, Messina, Italy; 14 Neurology Unit ,San Filippo Neri Hospital ASL Roma1, Rome, Italy; 15 Neurology Unit, Campus Bio-Medico University Hospital Foundation, Rome, Italy; 16 Unit of Neurology, Neurophysiology, Neurobiology, Department of Medicine, Università Campus Bio-Medico Di Roma, Rome, Italy; 17 Brain Innovations Lab, Università Campus Bio-Medico Di Roma, Rome, Italy; 18 Neurology Unit, Rovereto Hospital, APSS Trento, Rovereto, TN Italy; 19 Neurosciences Research Centre, Molecular and Clinical Sciences Institute, St. George’s, University of London, London, UK; 20 Parkinson’s Disease and Movement Disorders Unit, IRCCS Mondino Foundation, Pavia, Italy; 21 Department of Neurosciences and Reproductive and Odontostomatological Sciences, Federico II University, Naples, Italy; 22 Neurology Department, S. Chiara Hospital, Trento, Italy; 23 Neurology Department, S.Eugenio Hospital, Rome, Italy

**Keywords:** Dystonic tremor, Prevalence, Rest tremor, Essential tremor, Classification

## Abstract

**Introduction:**

The recently released classification has revised the nosology of tremor, defining essential tremor (ET) as a syndrome and fueling an enlightened debate about some newly conceptualized entities such as ET-plus. As a result, precise information of demographics, clinical features, and about the natural history of these conditions are lacking.

**Methods:**

The ITAlian tremor Network (TITAN) is a multicenter data collection platform, the aim of which is to prospectively assess, according to a standardized protocol, the phenomenology and natural history of tremor syndromes.

**Results:**

In the first year of activity, 679 patients have been recruited. The frequency of tremor syndromes varied from 32% of ET and 41% of ET-plus to less than 3% of rare forms, including focal tremors (2.30%), task-specific tremors (1.38%), isolated rest tremor (0.61%), and orthostatic tremor (0.61%). Patients with ET-plus were older and had a higher age at onset than ET, but a shorter disease duration, which might suggest that ET-plus is not a disease stage of ET. Familial aggregation of tremor and movement disorders was present in up to 60% of ET cases and in about 40% of patients with tremor combined with dystonia. The body site of tremor onset was different between tremor syndromes, with head tremor being most commonly, but not uniquely, associated with dystonia.

**Conclusions:**

The TITAN study is anticipated to provide clinically relevant prospective information about the clinical correlates of different tremor syndromes and their specific outcomes and might serve as a basis for future etiological, pathophysiological, and therapeutic research.

## Introduction

Tremor is deemed to be the commonest movement disorder. A population study performed in Northern Italy found tremor syndromes to be the most frequent movement disorder with a prevalence of 14.5% in people aged > 50 years, followed by restless legs syndrome (10.8%) and parkinsonism (6.95%) [1]. Different disorders can present with tremor and they span from very common conditions, including enhancement of physiological tremor (EPT), which is usually transient and non-symptomatic [2], to rare forms of tremor [3]. Probably being the commonest form of tremor seen in clinical practice, Essential Tremor (ET) has an estimated prevalence of 1% of the general population and has been formerly construed to be a mono-symptomatic condition with an autosomal dominant pattern of inheritance and characterized by a slow progression of tremor intensity with age [4]. Despite its relative frequency, research efforts into the identification of key pathophysiologic markers and of a defined genetic etiology have been mostly inconclusive [5]. This probably owes to the fact ET has been over-diagnosed with the inclusion of patients in whom other clinical features, such as dystonia, were missed as well as of patients with other forms of tremor (i.e., isolated tremors of the head/voice or even orthostatic tremor), which are instead likely driven by a different pathophysiology [5, 6].

Following this uncertainty, in 2018, the Tremor Task Force of the International Parkinson’s and Movement Disorders Society (IPMDS) published a new tremor classification [7], the structure of which is based on two axes: clinical features (axis I) and etiology (axis II). Accordingly, ET has been re-conceptualized as a clinical syndrome (axis I), rather than as a single disease entity, consisting of an isolated bilateral action tremor of the upper limbs with a duration of at least 3 years [7]. Furthermore, the construct of “ET-plus” was introduced for those patients fulfilling the criteria of ET but also having either a rest tremor or additional “soft signs” that do not suffice to make an alternative diagnosis [7]. Compared to previous definitions of ET, recent studies have suggested that only 15 [8] to 50% [9] of patients would be classified as ET with all the remaining showing additional soft signs and therefore fulfilling the criteria of ET-plus. The high discrepancy between these figures probably owes to the retrospective nature of these studies and therefore precise frequency estimates of ET, ET-plus, and other tremor syndromes are currently unknown. Similarly, precise information of demographics, clinical features, and about the natural history of these conditions are lacking and are highly warranted. In view of the new classification that has revised the nosology of tremor syndromes [7], a longitudinal multicenter collection of accurate and reliable clinical information would increase our understanding of these conditions.

Here, we describe The ITAlian tremor Network (TITAN), a multicenter data collection platform, the aim of which is to prospectively assess the phenomenology and natural history of tremor syndromes and to serve as a basis for future etiological, pathophysiological, and therapeutic research. The TITAN study is also likely to facilitate the dissemination and implementation of the new classification of tremor [7], which is crucial to harmonize the diagnosis of tremor syndromes across centers and to ensure correct recruitment of patients in dedicated clinical trials. In this work, we present the study design, methods and preliminary findings obtained from a large cohort of patients with tremor upon their baseline assessment.

## Methods

### Study design

The TITAN study has been proposed by the Department of Medicine, Surgery and Dentistry “Scuola Medica Salernitana”, Neuroscience section, University of Salerno, Baronissi, Italy, and approved by the ethic committee of the coordinator center (study approval n.33_r.p.s.o._02/10/2020). In the first year of activity, 30 movement disorder centers distributed throughout Italy have adhered to the TITAN study.

The TITAN study consists of two phases: (1) a transversal phase aimed to assess the frequency and clinical correlates of different tremor syndromes; and (2) a longitudinal phase consisting of annual follow-up visits aimed to assess the natural history of different tremor syndromes. The longitudinal phase duration is set to 10 years.

A virtual investigator meeting was held on January 2021 to recapitulate inclusion criteria, discuss the new tremor classification, explain the study related activities and standard operating procedures, and discuss potential sources of data errors. The registration of the meeting is made available to all participating sites. Patients’ information are recorded into a web-based encrypted anonymised system within the web site of the Fondazione Limpe per il Parkinson ONLUS (http://www.fondazionelimpe.it/) that promoted the study and is only responsible for data handling and the maintenance of the online portal, which complies with the General Data Protection Regulation. The principal investigator of the study (RE) is responsible for the conduct and reporting of the research project and for managing, monitoring, and ensuring the integrity of any collaborative relationships. Sharing the deidentified dataset will be considered on a case-by-case basis, upon reasonable request addressed to the principal investigator (rerro@unisa.it).

### Inclusion criteria

All subjects aged > 18 years with any tremor syndromes but the ones in the context of a clinical diagnosis of parkinsonism (i.e., presence of bradykinesia with either rest tremor or rigidity) [10] will be recruited upon signature of a dedicated consent form. Informed consent is an unconditional prerequisite for patient participation in the study, and data protection and privacy regulations are observed in capturing, forwarding, processing, and storing participant data. Participants are free to withdraw from the study at any time; unless otherwise requested by the participant, all data obtained up to that point will be retained.

### Core evaluations

Core assessments include the collection of demographic data (sex and age at evaluation), family history for tremor or any other neurologic disorders, age at onset, tremor distribution at onset, task-specificity at onset, presence of sensory-trick or position-dependence at evaluation, diagnosis according to the 2018 IPMDS classification of tremor, presence of “soft signs” (a free text tab is present on the portal to specify the soft sign(s)) or of associated features, and information about eventually performed imaging studies. Moreover, core assessments include the Essential Tremor Rating Assessment Scale (TETRAS) [11]. Since TETRAS does not capture rest tremor and in order to homogenize comparisons among different tremor syndromes including ET-plus that might in fact present with a rest component, an item assessing rest tremor was added to the scale. It scores rest tremor identically to action tremor (i.e., from 0 = no tremor to 4 = tremor is > 20-cm amplitude) during two different conditions (sitting and walking). Moreover, in order to assess the influence of motor patterns on tremor upon drawing the Archimedes spirals, the relative item was duplicated to have subjects drawing the spirals clockwise and anti-clockwise for each hand. Because of these implementations, we refer to this scoring tool as modified TETRAS (mTETRAS).

Finally, core assessments include the scale for the assessment and rating of ataxia (SARA) [12], the Quality of Life in Essential Tremor Questionnaire (QUEST) [13], and the EuroQol-5D instrument and a collection of previous/current treatments for tremor along with patient-reported outcomes according to the Clinical Global Impressions (CGI) Scale-Improvement (CGI-I).

### Ancillary evaluations

Optional evaluations include the MOntreal Cognitive Assessment (MOCA) [14], the Hospital Anxiety and Depression Scale (HADS) [15], and a customized questionnaire to assess the presence of prodromal symptoms of Parkinson’s disease [16]. Moreover, optional assessments include a video recording of the core-evaluation, an electrophysiologic study, and a collection of a blood sample for future genetic studies.

It is possible for all participating sites to propose ancillary studies that will be discussed and eventually approved by the scientific board of the TITAN study, with the relative assessment procedures/instruments made available on the online platform.

### Analysis of baseline demographic and clinical characteristics of the enrolled population

Statistical analysis herein presented has been performed by STATA 11 package using descriptive statistics (*t*-test, chi-squared test, one-way ANOVA, and post hoc tests as appropriate); *p* < 0.05 deemed as significant. Data were expressed as mean and standard deviation (SD) unless otherwise indicated.

## Results

By 31 January 2022, 679 patients were recruited. For 12 patients (1.77%), there were missing data precluding the correct diagnostic allocation and 14 patients (2.06%) were diagnosed with tremor combined with parkinsonism, which represents an exclusion criterion: these records were therefore excluded, leaving a sample of 653 patients (348 males and 305 females) with a mean age (± SD) at evaluation of 67.63 + 12.25 years that is herein described.

ET-plus represented the most common diagnosis (41.34%), followed by ET (32.01%) and combined tremors (14.55%). Among the latter, 89 patients (93.68%) had tremor combined with dystonia (i.e., including both dystonic tremors and tremor associated with dystonia, definitions that are based on the relative distribution of dystonia with respect of tremor, as per consensus [7]), whereas in 3 patients (3.15%), it was associated with ataxia or with a complex epileptic syndrome each. The breakdown of diagnostic allocations is depicted in Table 1, whereas Table 2 details the soft signs in patients with ET-plus.

**Table 1:** Breakdown of tremor syndromes in the entire cohort

Diagnosis	*N* (%)
ET-plus	270 (41.34%)
ET	209 (32.01%)
Combined tremors	95 (14.55%)
Isolated segmental action tremors	19 (2.1%)
Focal tremors	15 (2.30%)
Indeterminate tremor	10 (1.53%)
Task-specific tremors	9 (1.38%)
Enhanced physiologic tremor	9 (1.38%)
Isolated rest tremor	4 (0.61%)
Orthostatic tremor	4 (0.61%)
Other tremors (including functional tremor, neuropathic tremor, tremor with spasticity and tremor in multiple sclerosis)	9 (1.38%)

**Table 2:** Frequency of the soft signs in ET-plus

Soft signs	*N* (%)
Rest tremor	139 (51.48%)
Questionable dystonia	32 (11.85%)
Slowing	26 (9.63%)
Impaired tandem gait	14 (5.18%)
Subjective cognitive issues	13 (4.81%)
More than one soft sign	46 (17.03%)

Preliminary descriptive analyses have been performed to compare the three most prevalent tremor syndromes, namely ET, ET-plus and tremor combined with dystonia (Table 3). Whereas sex distribution was relative homogenous in ET and ET-plus, a higher proportion of females were found to have tremor combined with dystonia (*χ*2 = 14.91; *p* < 0.01; Table 3). These patients were younger than both ET and ET-plus, and the latter were the oldest between the three groups (*F* = 7.19; *p* < 0.01; Table 3). Significant differences in terms of age at onset were also found between the three groups (*F* = 5.89; *p* = 0.05; Table 3) with ET-plus  having the higher age at onset, whereas disease duration was longer in ET than in the other two groups (*F* = 6.89; *p* < 0.01). Family history for either tremor (*χ*2 = 17.03; *p* < 0.01; Table 3) or any movement disorders (*χ*2 = 11.11; *p* < 0.01; Table 3) was much more commonly reported by ET and ET-plus patients than patients with tremor combined with dystonia. Head tremor at onset was reported in a minority of patients with ET and ET-plus as compared to about 20% of patients with tremor combined with dystonia (*χ*2 = 46.67; *p* < 0.01; Table 3).

**Table 3:** Comparisons of the main demographic and clinical features between the three most common tremor syndromes

	ET	ET-plus	Tremor combined with dystonia*	*p*
Sex [male/female; *N* (%)]	122 (58.37%)/87 (41.63%)	119 (44.07%)/151 (55.93%)	33 (37.07%)/56 (62.93%)	< 0.001
Age (years; mean ± SD)	67.63 ± 12.26^a,c^	69.92 ± 11.06^b,c^	65.04 ± 12.46^a,b^	0.001
Age at onset (years; mean ± SD)	47.23 ± 22.56^a^	53.80 ± 19.23^b^	48.77 ± 20.18^a^	0.05
Disease duration (years; mean ± SD)	20.39 ± 20.17^a,c^	16.12 ± 16.31^b^	16.26 ± 16.07^b^	0.027
Family history for any movement disorders^+^ [yes/no; *N* (%)]	102 (62.96%)/ 60 (37.04%)	113 (54.58%)/94 (45.42%)	28 (39.43%)/43 (60.57%)	0.004
Family history for tremor^+^ [yes/no; *N* (%)]	77 (47.53%) / 85 (52.47%)	92 (44.45%)/115(55.55%)	14 (19.71%)/57 (80.29%)	< 0.001
Arm involvement at onset [*N* (%)]^				
-No -Unilateral -Bilateral symmetric -Bilateral asymmetric	-4 (1.92%) -48 (23.08%) -78 (37.05%) -78 (37.05%)	-12 (4.49%) -81 (30.33%) -80 (29.97%) -94 (35.21%)	-14 (20.29%) -26 (37.69%) -12 (17.39%) -17 (24.63%)	< 0.001

^*^Including both dystonic tremor and tremor associated with dystonia (see text for details). + Missing values: 47 (ET); 63 (ET-plus); 18 (tremor associated with dystonia). ^1 missing value in ET and 3 in ET-plus, whereas 20 patients with "tremor combined with dystonia" without arm involvement of tremor at the time of the evaluation were excluded from this analysis. ^a^Different from ET-plus; post hoc *p* < 0.05. ^b^Different from ET; post hoc *p* < 0.05. ^c^Different from tremor associated with dystonia; post hoc *p* < 0.05

## Discussion

Although a number of works [8, 9, 17–22] have been published after the release of the new tremor classification [7], most of which focused on the re-classification of formerly diagnosed patients with ET, the data herein presented provide the first accurate overview of different tremor syndromes, based on the baseline cross-sectional analysis of a prospective, multi-center assessment of patients with tremor.

Frequency figures of tremor disorders with regard to ET and ET-plus are largely in agreement with previously published studies [8, 9, 17–22], confirming the suggestion that ET-plus is more common than ET. Previous studies reported ET-plus frequency to range between about 50 to 85% of “ET” cases [8, 9, 17–22]. This large range across studies might be due to two main reasons: (1) the recruitment in tertiary movement disorder centers, which might have led to selection biases [23]; and (2) the fact that all these studies had a retrospective design, attempting a diagnostic reclassification based on medical record review. Our result of ET-plus representing about 56% of “ET cases” conservatively places at the lower boundary of the reported range and might be more representative of the entire population of ET-like tremors because one of the strengths of the TITAN study stands in its multi-center design, which involves both secondary and tertiary movement disorder centers and thus minimizes to some extent the risk of recruitment bias. Moreover, the figures here provided are based on the assessment of patients following the new tremor classification. Of note, this is the first study providing relative frequency of tremor syndromes beyond ET and ET-plus, therefore including rarer forms including isolated segmental action tremors, focal and task-specific tremors, and orthostatic tremor. It should be also noted that the commonest form of tremor (i.e., EPT) was found in less than 2% of our patients: this result, which would seem to contrasts with the conception that EPT is the commonest tremor [1], is easily explained by the fact that this form of tremor is usually non-symptomatic and subjects with EPT do not generally seek medical advice.

When looking at the types and frequency of soft signs associated with ET-plus, rest tremor was found to be largely the commonest (about 50% of cases), followed by questionable dystonia (about 11%) and undetermined slowing (about 9% of cases). These results are broadly in agreement with the majority of studies [17, 21, 22] but not with Pandey and Bhattad [19], who in their prospective assessment of ET-plus cases found rest tremor in only 2 out of 45 ET-plus cases (4.44%). The latter result might be due to the single-center recruitment as well as to the specific expertise of the raters (focused on dystonia) [24], limitations that are arguably minimized in the TITAN study because of its multi-center design, as mentioned above.

Sex distribution of ET (and ET-plus) is in line with previous findings [4], as it is for tremor combined with dystonia [25], which was found to be more common in females, recapitulating sex distribution of adult-onset dystonia in general [26].

Interestingly, a positive family history, particularly but not only for tremor, was found in about 50–60% of cases with ET and ET-plus which reinforces the concept of a genetic susceptibility in both syndromes, despite the largely negative efforts pursued in the past to find a genetic cause of ET [5]. Conversely, family history for tremor was much less common in patients with dystonia, although it was present in about 20% of cases. Given the self-report of family history, it is impossible to ascertain whether in these family members tremor was present in addition to dystonia or was an isolated finding. The latter hypothesis would support the concept of dystonia being a phenotypic continuum between abnormal posturing and tremor [27], with the two features being associated in some cases. Beyond this speculation, our results remark on the fact that tremor is part of the phenotypic spectrum of dystonia and therefore, dystonia should be carefully looked for when assessing tremulous patients to avoid misdiagnosis [28].

Of note, patients with ET-plus were older and had a higher age at onset than ET cases. Conversely, the latter had longer disease duration than the former. These results are in contrast with Louis et al. who found ET-plus cases to be older than ET as a function of longer disease duration, and therefore suggested ET-plus being a “disease stage” of ET [17]. Our results do not support this proposal and would rather suggest that ET-plus represents a group of different entities, which, at least in cases with onset in the elderly, might be linked to pathological aging and arguably less dependent on genetic predisposition [29].

Another novelty of the new tremor classification is the removal of patients with isolated focal tremor of the head and voice from the rubric of ET [7]. However, our results show that tremor at onset might involve other body regions beyond the arms in a minority of patients with ET and ET-plus. Therefore, although head tremor is more common in dystonia and patients with isolated head tremor are more likely to develop overt dystonia during the disease course [30], this would not always be the rule. Longitudinal assessments of patients with isolated focal tremors of the head or voice in this study will eventually clarify whether there are clinical features predicting the final diagnostic allocation.

We acknowledge some limitations. We cannot entirely exclude a recruitment bias that is inherent to studies without a population-based design. However, the inclusion of both secondary and tertiary movement disorder centers might have, at least in part, attenuated this risk and therefore might provide frequency figures of tremor syndromes that should be more realistic than those obtained in single-center studies. Moreover, we acknowledge that the diagnosis of tremor syndromes is made on clinical basis given the lack of available biomarkers, and this might carry the risk of misdiagnosis in a proportion of patients. However, we strictly adhered to the current classification [7], which does not require any additional testing for the formal definition of the proposed tremor syndromes [6]. Finally, there are no operational criteria for the definition of “soft signs” and their interpretation is, per consensus, subjective and left to the investigator [7]. This might clearly represent a source of ambiguity [31]. However, we note that (1) the frequency of ET-plus herein reported is in line with previous studies and (2) rest tremor, which was the most frequent soft sign in this as well as in many other studies, does not strictly represent a finding “of uncertain relationship to tremor” [7], thus minimizing the ambiguity regarding the highly debated construct of ET-plus [32].

In summary, we have here presented the rationale and design of the TITAN study, the preliminary results of which can already inform about the relative frequency and main clinical features of the newly conceptualized tremor syndromes. The TITAN study is anticipated to provide clinically relevant prospective information about the clinical correlates of different tremor syndromes, their specific outcomes, and the eventual transition across different diagnostic allocations and also to generate hypotheses for future investigations.
