# Correction to: The Italian tremor Network (TITAN): rationale, design and preliminary findings

**DOI:** 10.1007/s10072-022-06216-3

**Published:** 2022-06-20

**Authors:** Roberto Erro, Andrea Pilotto, Marcello Esposito, Enrica Olivola, Alessandra Nicoletti, Giulia Lazzeri, Luca Magistrelli, Carlo Dallocchio, Roberta Marchese, Matteo Bologna, Alessandro Tessitore, Salvatore Misceo, Angelo Fabio Gigante, Carmen Terranova, Vincenzo Moschella, Lazzaro di Biase, Raffaella Di Giacopo, Francesca Morgante, Francesca Valentino, Anna De Rosa, Assunta Trinchillo, Maria Chiara Malaguti, Livia Brusa, Angela Matinella, Francesca Di Biasio, Giulia Paparella, Rosa De Micco, Elena Contaldi, Nicola Modugno, Alessio Di Fonzo, Alessandro Padovani, Paolo Barone, TITAN Study Group

**Affiliations:** 1 Department of Medicine, Surgery and Dentistry “Scuola Medica Salernitana”, Neuroscience Section, University of Salerno, Via Allende 43, 84081 Baronissi, SA Italy; 2 Neurology Unit, Department of Clinical and Experimental Sciences, University of Brescia, Brescia, Italy; 3 Clinical Neurophysiology Unit, AORN Cardarelli, Naples, Italy; 4 Neuromed Institute IRCCS, Pozzilli, IS Italy; 5 Department “G.F. Ingrassia”, Section of Neurosciences, University of Catania, Catania, Italy; 6 Neurology Unit, Department of Neuroscience, Dino Ferrari Center, Fondazione IRCCS Ca’ Granda Ospedale Maggiore Policlinico, Milan, Italy; 7 Department of Translational Medicine, Movement Disorders Centre, Neurology Unit, University of Piemonte Orientale, Novara, Italy; 8 Neurology Unit, Department of Medical Area, ASST Pavia, Voghera, PV Italy; 9 IRCCS Ospedale Policlinico San Martino, Genoa, Italy; 10 Department of Human Neurosciences, Sapienza University of Rome, Rome, Italy; 11 Department of Advanced Medical and Surgical Sciences, Università Della Campania “Luigi Vanvitelli”, Naples, Italy; 12 Neurosensory Department, Neurology Unit, San Paolo Hospital, ASL Bari, Bari, Italy; 13 Department of Clinical and Experimental Medicine, University of Messina, Messina, Italy; 14 Neurology Unit ,San Filippo Neri Hospital ASL Roma1, Rome, Italy; 15 Neurology Unit, Campus Bio-Medico University Hospital Foundation, Rome, Italy; 16 Unit of Neurology, Neurophysiology, Neurobiology, Department of Medicine, Università Campus Bio-Medico Di Roma, Rome, Italy; 17 Brain Innovations Lab, Università Campus Bio-Medico Di Roma, Rome, Italy; 18 Neurology Unit, Rovereto Hospital, APSS Trento, Rovereto, TN Italy; 19 Neurosciences Research Centre, Molecular and Clinical Sciences Institute, St. George’s, University of London, London, UK; 20 Parkinson’s Disease and Movement Disorders Unit, IRCCS Mondino Foundation, Pavia, Italy; 21 Department of Neurosciences and Reproductive and Odontostomatological Sciences, Federico II University, Naples, Italy; 22 Neurology Department, S. Chiara Hospital, Trento, Italy; 23 Neurology Department, S.Eugenio Hospital, Rome, Italy

**Correction to: Neurological Sciences (2022)**

https://doi.org/10.1007/s10072-022-06104-w

Originally, the article was published with an error. The affiliation of the author Giulia Paparella should only be " Neuromed Institute IRCCS, Pozzilli, IS, Italy”.

The original article has been corrected.

